# Effects of Gen-AI integrated science reading activity on primary school students’ intrinsic reading motivation

**DOI:** 10.3389/fpsyg.2026.1865488

**Published:** 2026-08-19

**Authors:** Xueshen Wang, Shasha Tian, Yun Wei

**Affiliations:** 1 Faculty of Education, Ningxia Normal University, Guyuan, China; 2 Faculty of Education, Languages, Psychology, and Music, SEGI University, Kota Damansara, Malaysia

**Keywords:** Gen-AI, intrinsic reading motivation, mixed method, primary school students, science reading activity

## Abstract

**Introduction:**

Generative Artificial Intelligence (Gen-AI) can generate new content such as text, images, audio, and video based on users’ needs. This study designed a Gen-AI integrated science reading activity to examine its effects on primary school students’ intrinsic reading motivation.

**Methods:**

A mixed research design was adopted with sixth-grade students from a public primary school in central China. Two classes were randomly assigned to an experimental group (Gen-AI integrated reading) and a control group (traditional reading).

**Results and Discussion:**

Independent-samples t-tests showed no significant differences between the two groups in intrinsic reading motivation before the experiment. After the intervention, the experimental group showed greater improvement than the control group. ANCOVA results indicated that the Gen-AI integrated science reading approach had significant positive effects on intrinsic reading motivation. Semi-structured interviews further revealed that the approach can satisfy students’ autonomy, competence, and relatedness from multiple aspects. In addition, the limitations and future prospects of this study were also discussed.

## 1 Introduction

As a special form of subject reading, science reading not only carries the transmission of science knowledge, but also undertakes the task of cultivating students’ science literacy, enhancing their understanding and exploration ability of science concepts (Schiefer et al., 2020). Therefore, conducting science reading activities in school science teaching is not only a powerful supplement to students’ science learning, but also an important way to cultivate their conceptual construction, evidence evaluation, and exploration abilities (Cooper et al., 2022; Koray and Çetinkılıç, 2020). Compared with general reading, science reading texts often have the characteristics of abstract concepts, stringent chains of reasoning, dense discipline-specific terminology, and the integration of multimodal information (e.g., figures, data, and experimental procedures), which puts higher cognitive load on students in understanding and transferring (Brüggemann et al., 2023; Liu, 2024; Mikk and Kukemelk, 2010). Therefore, how to design effective science reading activities that support students’ cognitive understanding while improving their intrinsic reading motivation is an issue worth exploring in science education research.

Generative Artificial Intelligence (Gen-AI) is a specialized branch of AI that focuses on creating new content based on individual needs, such as text, images, audio, and video (Brindha et al., 2025). Unlike traditional AI systems that primarily recognize patterns and make predictions, Gen-AI actively generates analysis results based on user prompts and delivers corresponding outputs. Therefore, Gen-AI overcomes the shortcomings of traditional AI, such as low efficiency, high cost, low quality, and high subjectivity, and to some extent improves the efficiency and quality of AI-driven recognition and analysis (Bandi et al., 2023; Bommasani et al., 2021). In addition, through continuous learning and optimization in the process of data recognition and analysis, the generalization ability of Gen-AI models will gradually improve (Pescapè, 2024; Ravichandran et al., 2023).

Given the enormous potential of Gen-AI, many educators attempt to integrate it into educational and teaching activities to better assist students in learning (Sun and Zhou, 2024). At present, the use of Gen-AI to enhance the science reading intrinsic motivation of primary school students is still in its infancy, and lacks relevant empirical research. In this study, we designed a Gen-AI integrated science reading activity and we hope that this research can provide reference for the future research and application of Gen-AI in science education in primary schools. Specifically, this study aimed to explore the following questions:

1. Can the Gen-AI integrated science reading activity improve primary school students’ intrinsic reading motivation in comparison with traditional science reading activity?
2. How does Gen-AI integrated science reading activity improve primary school students’ intrinsic reading motivation?

## 2 Literature review

### 2.1 Gen-AI and science reading

In recent years, the rapid development of Gen-AI has provided a new way for science reading activity. Unlike traditional AI-assisted learning systems, Gen-AI can interact with students in a conversational manner and generate personalized explanations based on their specific questions. This feature makes Gen-AI particularly suitable for supporting cognitive and metacognitive needs in the process of science reading.

Combining the functions of Gen-AI, it can improve the science reading activity in the following ways: firstly, Gen-AI can provide real-time explanations when students read science texts. If students encounter unfamiliar terms or abstract concepts while reading, they can request examples, analogies, or visual descriptions from Gen-AI to reduce cognitive load and maintain reading fluency (Dewi et al., 2025). Secondly, Gen-AI can generate diverse hypotheses and perspectives. Before or during science reading, Gen-AI can generate multiple possible explanations based on phenomena, inspiring students to read with questions and transforming reading from passive information reception to an active verification process (Jiang, 2026). Thirdly, Gen-AI can provide role-playing and conversational functions. By allowing teachers to input relevant instructions, Gen-AI can play the role of the scientist in reading texts and engage in virtual conversations with students, thereby enabling students to understand the historical context and social context in which science knowledge is generated, enhancing the situational and emotional participation of reading (Dai et al., 2025). Finally, Gen-AI can support students in conducting independent questioning and exploration. Unlike traditional reading activity with pre-set questions, Gen-AI allows students to ask questions based on their curiosity and understanding difficulties, and receive immediate, non judgmental responses, which helps cultivate students’ problem awareness and inquiry skills (He and Lu, 2024).

Previous studies have preliminarily confirmed that AI can enhance students’ intrinsic reading motivation in reading activities. Hidayat (2024) found that humanized reading activities based on AI reading platforms indirectly enhance students’ intrinsic reading motivation by matching their reading levels. Alfaleh et al. (2025) conducted a quantitative study on 297 EFL students, which showed a significant positive correlation between the participation of Gen-AI tools such as ChatGPT and students’ intrinsic motivation for oral reading. However, most of these studies focus on language or general reading, and research on the specific context of science reading is still limited. The particularity of science reading means that the influence mechanism of Gen-AI may be different from general reading and requires specialized research to explore.

### 2.2 Intrinsic reading motivation

Reading motivation refers to the driving force or reasons that motivate individuals to read. It includes attitudes, values, and behaviors that affect individual reading frequency and effectiveness (Guthrie and Coddington, 2009; Schiefele et al., 2012). Reading motivation is a key factor in promoting the development of individual reading abilities, as motivated readers tend to read more frequently, which enhances their reading skills and comprehension abilities (Ahmadi, 2017). Reading motivation can be divided into intrinsic motivation and extrinsic motivation (Troyer et al., 2019). Intrinsic motivation is driven by an individual’s interest, enjoyment, curiosity, or satisfaction with reading content. Intrinsic motivation drives readers to engage in reading not for the purpose of obtaining external rewards, but rather because they feel that the content of the reading material is meaningful and that they can gain a pleasurable experience through reading (Li and Gan, 2022). Extrinsic motivation is the use of external factors such as grades, rewards, recognition, or avoidance of negative consequences to drive individuals to read. Although extrinsic motivation can initially motivate individuals to actively engage in reading, its long-term effects are not as long-lasting as intrinsic motivation, therefore, educators should focus on cultivating students’ intrinsic motivation for reading (Ramalingam and Jiar, 2022).

In science reading activity, intrinsic reading motivation plays an important role. Due to the fact that science texts often contain a large amount of new information, abstract concepts, and logical chains, students need to invest sustained cognitive effort to understand them. Students with higher intrinsic reading motivation are more likely to persist in reading, actively seek explanations instead of giving up when encountering difficulties, and extend science reading beyond the classroom (Duke et al., 2021; Wang et al., 2022). However, research indicates that students’ intrinsic reading motivation of science texts tends to decline with age. Many students perceive science texts as “boring” or “difficult,” a phenomenon partly attributable to traditional science reading activities that overemphasise factual memorisation while neglecting students’ curiosity, sense of choice, and knowledge construction processes (Schiefer et al., 2020). Therefore, improving traditional science reading activities has become the key to enhancing students’ intrinsic motivation for science reading.

### 2.3 Theoretical framework

In this study, we drew on the self-determination theory as theoretical foundation. Self-determination theory stated that the autonomy and initiative of human behavior stem from the fulfillment of three basic psychological needs: autonomy, competence, and relatedness. When learning situations adequately support these three psychological needs, students will demonstrate high levels of intrinsic motivation, sustained cognitive engagement, and positive behavior (Deci and Ryan, 2013; Ryan et al., 2021). Gen-AI can provide students with a highly flexible interactive experience. During science reading, students can ask Gen-AI questions based on their personal interests and independently select information they deem reasonable (Khlaif et al., 2025). This open learning environment enhances students’ sense of control and choice, satisfying their need for autonomy in learning. Gen-AI can provide students with diverse perspectives during discussions, broadens their understanding of reading content, and helps them better understand science concepts, thereby enhancing their competence in science learning (Chan and Hu, 2023). Gen-AI can not only provide knowledge-based feedback, but also simulate different roles to interact with students, enriching their emotional experience in science reading. In addition, by analyzing the answers provided by Gen-AI and sharing opinions with peers, students also gain emotional recognition and social support through group interaction (Huang and Lajoie, 2023). Therefore, Gen-AI satisfies students’ relatedness needs in science learning.

## 3 Method

This study employed an explanatory sequential mixed-methods design to investigate the effects of Gen-AI integrated science reading activity on primary school students’ intrinsic reading motivation. For questions 1, this study adopted quasi-experimental method. Quasi-experimental study is usually conducted in real environments, where researchers can observe phenomena that occur in real-life. This enhances the external validity of the research results, making them more applicable to a wider population and real-world situations (Campbell and Stanley, 2015). For question 2, this study used semi-structured interviews to collect qualitative data and explore the ways in which Gen-AI integrated science reading activity affect students’ intrinsic reading motivation.

### 3.1 Participants

Considering the difficulties faced by lower grade primary school students in using Gen-AI, this study was conducted among higher grade students. This study randomly selected two classes from the sixth grade of a public primary school in central China, with a total of 103 students (44 boys and 59 girls) as research subjects, aged between 11 and 12 years old (see Table 1). Students from two classes were assigned to the experimental group (Gen-AI integrated reading method) and the control group (traditional reading method). The science reading activities of two classes were organized by the same science teacher, who is well versed in the use of Gen-AI. In the Gen-AI integrated science reading activity, the teacher acted as a facilitator, scaffolding students’ interactions with the Gen-AI (for example, the teacher circulated among groups to ensure that students not only asked the Gen-AI but also critically discussed its answers, prompting group’s thinking with questions such as: Do you agree with the AI? Why or why not?) and orchestrating the overall learning process (for example, the teacher synthesised the key science ideas, clarified any remaining misconceptions). In addition, the reading content for both classes was the same.

**Table 1:** Demographic information.

Group	Gender		Total
	Boy	Girl	
Experimental group	21	30	51
Control group	23	29	52

According to the Declaration of Helsinki, this study strictly followed ethical standards and provided informed consent forms to all participants’ parents or guardians, and obtained written permission from them. Throughout the entire research process, participants were informed that they could withdraw from the study at any stage according to their own wishes without being penalized.

### 3.2 Intrinsic reading motivation scale

This study aimed to focus on the intrinsic motivation of primary school students towards science reading. The reading motivation scale developed by Wigfield and Guthrie (1997) has been translated into Chinese version and widely used to measure the reading motivation of Chinese primary and secondary school students. This study selected the intrinsic reading motivation section to measure students’ intrinsic motivation for science reading. This scale includes 9 items and utilizes a 5-point Likert scale (1 = strongly disagree, 5 = strongly agree).

The scale included three dimensions, the first dimension was reading curiosity, which referred to students are interested in the content of science reading and subsequently engage in it. It was measured through 3 items, such as: 1. When the teacher discusses a problem that interests me, I will read relevant content to learn more. The second dimension was reading involvement, which referred to the level of engagement and feelings of students in the process of science reading, measured through 3 items, such as: 4. When I read texts, I often find myself immersed in them, oblivious to the passing of time. The third dimension was importance of reading, which referred to students consider that science reading is important for their science learning, It was also measured through 3 items, such as: 7. Reading can help me gain a wealth of knowledge. The Cronbach’s alpha coefficient was 0.942, indicating that the scale had good internal consistency.

### 3.3 Procedure

**Figure 1:**
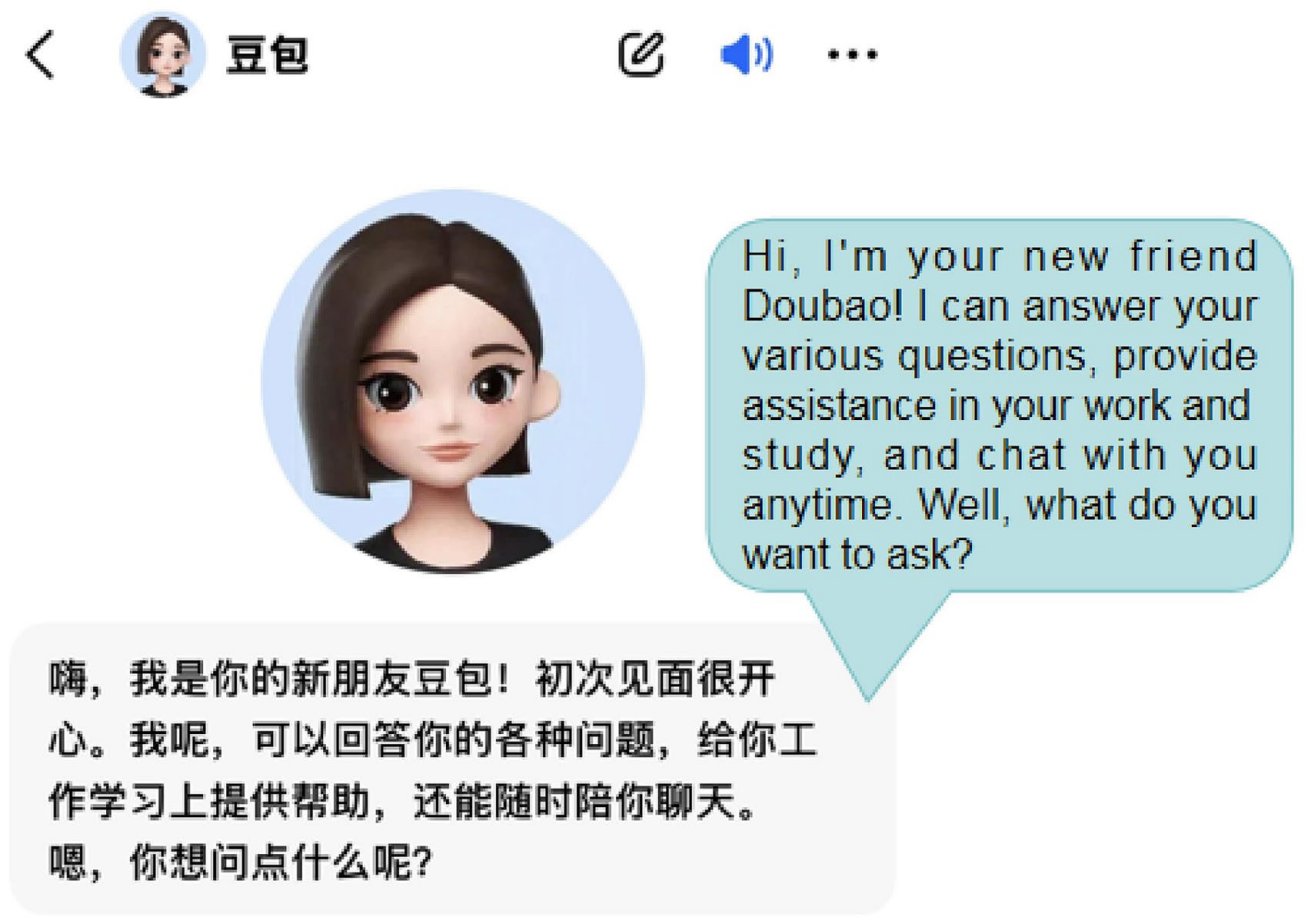
Doubao. Screenshot of the Doubao (ByteDance) AI‑chatbot interface captured by the author.

Due to network restrictions, Gen-AI systems launched abroad such as ChatGPT cannot be used in China. Therefore, in the Gen-AI integrated science reading activity, this study chose *Doubao* (Figure 1) to assist students in reading. *Doubao*, as a Gen-AI tool which can be accessed and used for free, has a simple user interface, fast system response, and excellent problem analysis and explanation functions, making it suitable for primary school students to use in science reading activity.

*Doubao* can communicate with students through text, voice, and video, answer various questions they may have while reading science materials, and help them acquire relevant science knowledge. In addition, the intelligent agent function of *Doubao* can generate various virtual images of scientists and interact with students, creating a scene of dialogue between students and “scientists” across time and space (For example, teachers can pre-set in *Doubao*: “Please play Galileo. You live in Italy in the 1600s. You speak in the first person ‘I’, with a tone that sounds like a patient grandfather or a determined scientist. You feel wronged by the church for making you confess, but you still love science. Answer questions from sixth grade students.”).

**Figure 2:**
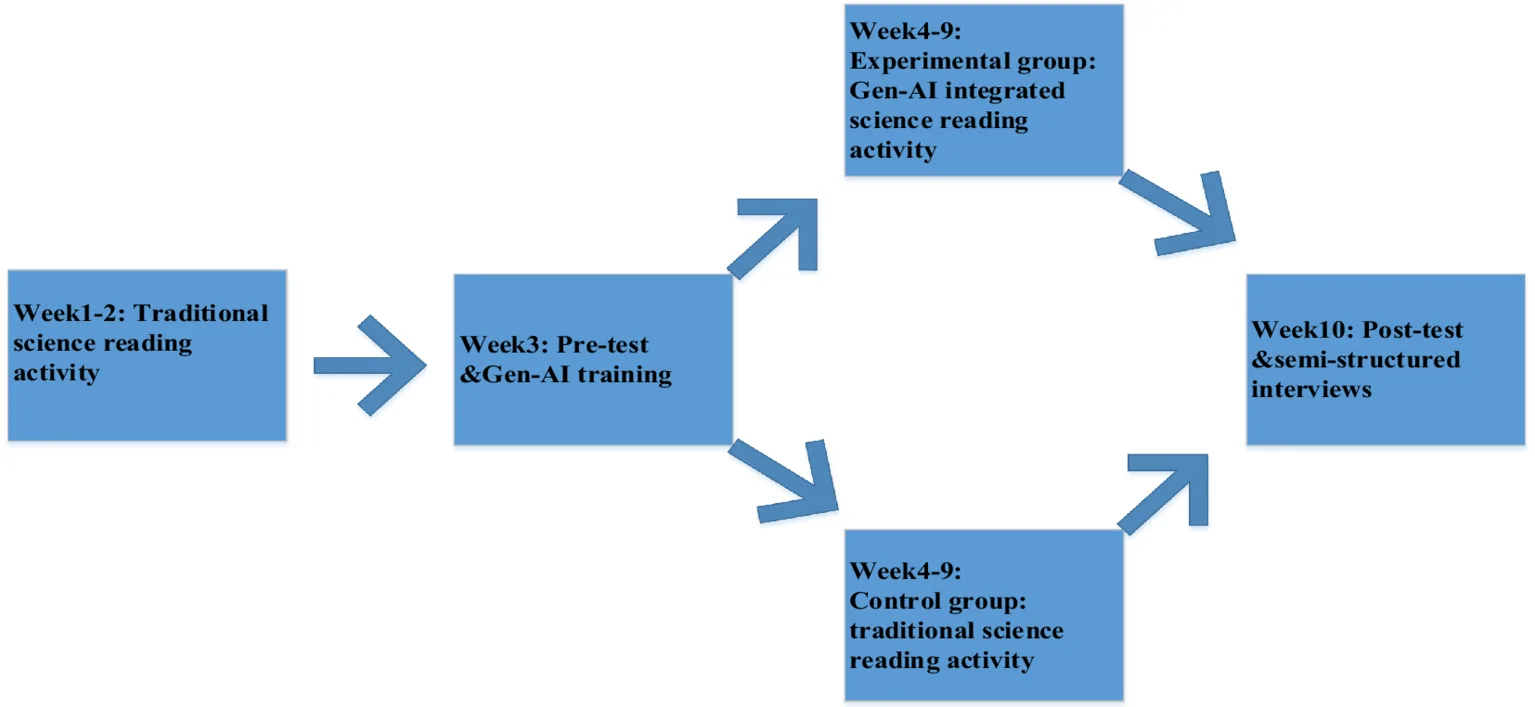
The experimental procedure.

Science reading activity was conducted once a week. The students in the control group engaged in science reading activities for 1 hour during their club time on Wednesday, while the experimental group engaged in science reading activities for 1 hour during their club time on Friday. Figure 2 showed the experimental procedure. In the first 2 weeks, both groups of students engaged in traditional science reading activity (see Table 2 for the activity process). In the third week, two groups of students underwent a 1 week pre-test on intrinsic reading motivation.

**Table 2:** Traditional science reading activity process.

Step	Activity	Time
1	Teacher showcases science phenomena through pictures or videos, inspiring students to think about the reasons behind the phenomenon.(For example, the teacher showed the picture of Galileo conducting a free fall experiment, and the students speculated on the results and reasons of the experiment.)	5 min
2	Students read relevant science materials. (For example, teachers organized students to read textual materials about Galileo’s research experience.)	15 min
3	The teacher presents questions related to the reading content, and students are divided into groups to discuss these questions. (For example, teachers may ask three questions about the text materials that students read: What were the results of Galileo’s free fall experiment? What phenomena did Galileo observe with a telescope? Why do these phenomena support the idea that the Earth revolves around the Sun?)	10 min
4	Each group presents and shares their own viewpoints, while the remaining groups evaluate these viewpoints. (For example, Group A reported that Galileo simultaneously threw two iron balls of different weights from the Leaning Tower of Pisa, which landed at the same time, proving Aristotle’s viewpoint wrong. Group B added that Galileo did not conduct the experiment at the Leaning Tower of Pisa, as the reading material provided by the teacher stated “legend.”)	15 min
5	Teacher summarizes and evaluates students’ viewpoints.	5 min
6	Students exchange their feelings about this reading content. (For example, the teacher guided students to take turns sharing their feelings after reading Galileo’s story. Some students believed that Galileo was very brave, daring to challenge the church, and so on.)	10 min

Before the experiment, the students in the experimental group were randomly divided into 10 groups (5–6 students per group), and each group member shared a tablet computer. Science teacher provided a 30 min training to the students in experimental group. They were required to be familiar with the functions and operating methods of *Doubao,* which helped to reduce the difficulties when students using the new technology. Over the next 4 to 9 weeks, students in the experimental group participated in Gen-AI integrated science reading activity (see Table 3 for the activity process), while students in the control group continued to engage in traditional science reading activity. After the experiment, all students were required to complete a post-test on intrinsic reading motivation.

**Table 3:** Gen-AI integrated science reading activity process.

Step	Activity	Time
1	Teacher showcases science phenomena through pictures or videos, and interacts with students using Gen-AI to generate different hypotheses, students choose a hypothesis they believe is correct. (For example, the teacher presented pictures of Galileo’s free fall experiment and asked Gen-AI to generate different hypotheses based on the question ‘who lands first when objects of different weights fall from the same height’. Students chose their most trusted hypothesis, and the teacher records the votes on the blackboard.)	5 min
2	Students read relevant science materials and record their interested questions during the reading process. (For example, teachers organized students to read textual materials about Galileo’s research experience, and required each student to write down at least 2 questions that they are truly curious about.)	15 min
3	Students ask Gen-AI questions about what they are interested in reading. Discussions are held in response to the answers given by Gen-AI, and group members select and summarize the ideas they think are reasonable. (For example, a student input: “Can Galileo’s telescope see aliens? Gen-AI answered:” Galileo’s telescope is very simple, it can only magnify about 20 times. When he looks at the moon, he can see craters, and when he looks at Jupiter, he can only see small dots of light. He definitely cannot see aliens. Even today, we have not found any aliens. “In response to the answer of Gen-AI, the group discussed and concluded:” Galileo’s tools limited his discoveries, but they were already very powerful.”)	10 min
4	Each group presents and shares their own viewpoints, while the remaining groups evaluate these viewpoints. (For example, Group A reported: “Can Galileo’s telescope see Saturn’s rings.” The answer given by AI is: “No, because his telescope is too blurry.” Other groups agreed with Group A’s viewpoint, while Group C added that Galileo discovered Saturn had ‘ears’ at the time.)	15 min
5	Teacher summarizes and evaluates students’ viewpoints	5 min
6	Students engage in virtual conversations with scientists mentioned in the reading content through Gen-AI. (For example, a student asks Gen-AI, “Mr. Galileo, are you afraid of death? Did you pretend to confess at that time?” Gen-AI replied, “Child, I read a confession letter because I did not want to be burned to death. But when I left the courtroom, I gently said, ‘But the earth is indeed moving.’ Scientific facts will not change just because of authoritative denial. I advise you: never give up the pursuit of truth out of fear, but also learn to protect yourself - these are the two sentences I can give you.”)	10 min

To avoid selection bias, seven students were randomly selected from the experimental group to form a focus group to discuss the differences in their experiences when participating in Gen-AI integrated science reading activity and in traditional science reading activity. Subsequently, the semi-structured interview was conducted with the science teacher who participated in this experiment, asking her about her views on Gen-AI integrated science reading activity. The interview outline was jointly written by two researchers, including opening questions, introductory questions, key questions, and closing questions (Krueger and Casey, 2015).

This study invited two science teachers with 8 years of teaching experience to evaluate the interview outline and made corresponding modifications based on their suggestions. Both teachers considered that the revised interview outline was consistent with the research objectives, and the questions expression method was in line with the understanding level of sixth grade students, which can be used for subsequent research (see Tables 4, 5).

**Table 4:** Interview outline of focus group.

Step	Example
Opening questions	Dear classmates, welcome to this discussion. Next, I will ask you some questions. Please answer truthfully according to your actual situation. Is everyone ready?
Introductory questions	In this semester, you used the Doubao system to help yourself complete science reading tasks. Compared to previous science reading activity, which method do you prefer?
Key questions	What assistance and experience did Doubao bring to you during the science reading activity? What influence have these assistance and experience had on your attitude towards science reading?
Closing questions	Do you have any additional information or explanations to provide regarding the answers to the above questions?

**Table 5:** Interview outline of semi-structured interview.

Step	Example
Opening questions	Hello, thank you for participating in this research. Next, I will ask you some questions. Please answer truthfully based on your actual situation. Are you ready?
Introductory questions	Do you think there is a difference in students’ attitudes towards science reading between two classes?
Key questions	What is your opinion on integrating Gen-AI technology into science reading activity?
Closing questions	Do you have any additional information or explanations to provide regarding the answers to the above questions?

### 3.4 Data analysis

Quantitative analysis was used in this study to address research question 1. Firstly, the data collected were tested for normal distribution to ensure that they followed a normal or approximately normal distribution, thus facilitating subsequent analysis. Secondly, descriptive statistics were performed on the data to obtain analysis results such as mean and standard deviation. In order to understand the general trend of the data, paired sample t-test was conducted. Thirdly, independent sample *t*-test was used to test whether there was a significant difference in the pre-test and post-test data between the experimental and control groups. Finally, the Analysis of Covariance (ANCOVA) was used to explore the effects of Gen-AI integrated reading methods on primary school students’ intrinsic reading motivation.

**Figure 3:**
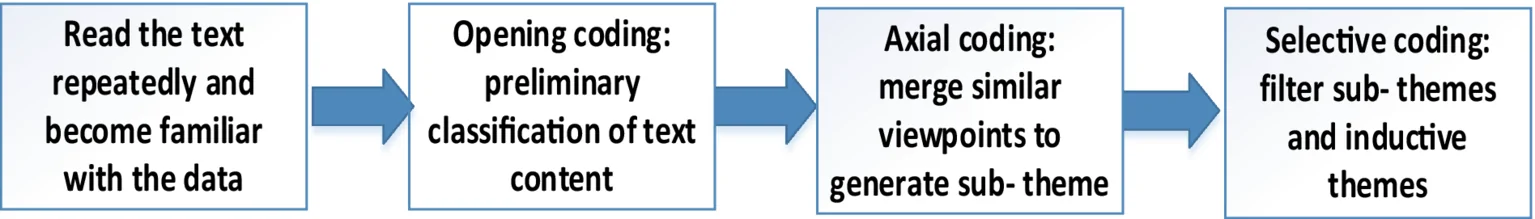
The coding scheme.

To address the research question 2, qualitative analysis was used. The semi-structured interview content was first transcribed into text, and the participating students and teacher were asked to check the text content to ensure consistency with what they express. Subsequently, thematic analysis was conducted on text content. This study used a three-level coding method to extract and inductive themes from the responses of students and teachers (Clarke and Braun, 2017). The coding scheme was shown in Figure 3.

To ensure coding accuracy and reliability, the coding in this study was completed by two researchers separately. Cohen’s kappa coefficient was used to analyze the consistency of the two researchers coding results. According to statistics, the coding consistency rate between two researchers was 75%, achieving significant consistency (Ismael et al., 2021). Then, the two researchers discussed their differences, reached a consensus, and completed the final codes. The coding result was reviewed by two science teachers, and both teachers acknowledged the result.

## 4 Results

### 4.1 Quantitative analysis

Kline (2023) stated that when skewness is within ± 3 and kurtosis is within ± 10, the sample data can be considered to follow a normal distribution. The skewness of the pre- and post-test data on students’ intrinsic reading motivation ranged from −0.957 to 0.347, and the kurtosis ranged from −0.658 to 1.127, indicating that the skewness and kurtosis values of the data met the criteria for normal distribution.

The descriptive statistical results of intrinsic reading motivation were shown in Table 6. For the experimental group, the pre-test mean of intrinsic reading motivation was 3.362, and the post-test mean was 3.882. Meanwhile, for the control group, the pre-test mean of intrinsic reading motivation was 3.325, and the post-test mean was 3.632. The results of the paired sample t-test indicated that both the Gen-AI integrated science reading activity and the traditional science reading activity significantly increased students’ intrinsic reading motivation (see Table 7).

**Table 6:** The descriptive statistical results of intrinsic reading motivation.

Test	Group	*N*	Mean	SD	Std. Error of mean
Pre-test	Experimental	51	3.362	0.918	0.129
	Control	52	3.325	0.884	0.123
Post-test	Experimental	51	3.882	0.405	0.057
	Control	52	3.632	0.451	0.063

**Table 7:** The paired sample *t*-test results of intrinsic reading motivation.

Group	Test	Mean	SD	Lower	Upper	t	df	Sig.
Experimental	Pre-test	3.362	0.918	0.240	0.801	3.730	50	0.000
	Post-test	3.882	0.405					
Control	Pre-test	3.325	0.884	0.070	0.545	2.599	51	0.012
	Post-test	3.632	0.451					

The results of the independent samples *t*-test were shown in Table 8, which manifested that the intrinsic reading motivation levels of the experimental and control groups were not significantly different in the pre-test and appeared significantly different in the post-test. This indicated that the experimental group’s intrinsic reading motivation increased significantly more than that of the control group.

**Table 8:** The independent sample *t*-test results of intrinsic reading motivation.

Test	t	df	Sig.	Mean difference	Std. Error difference	Lower	Upper
Pre-test	0.208	101	0.836	0.037	0.178	−0.315	0.389
Post-test	2.957	101	0.004	0.250	0.085	0.082	0.418

In order to assess the extent to which different reading activities affect students’ intrinsic motivation to read, the ANCOVA was conducted. Intrinsic reading motivation post-test scores were used as the dependent variable, group was used as the independent variable, and intrinsic reading motivation pre-test scores were used as the covariate. Preliminary checks ensured that the assumptions of normality, homogeneity of regression slopes, and homogeneity of variances were met and the ANCOVA analysis results were shown in Table 9.

**Table 9:** The ANCOVA results of intrinsic reading motivation.

Source	Type III sum of squares	df	Mean square	F	Sig.	Partial Eta Squared (η2)
Corrected Model	2.181	2	1.091	6.061	0.003	0.108
Intercept	82.852	1	82.852	460.466	0.000	0.822
Pre-test	0.574	1	0.574	3.187	0.077	0.031
Group	1.568	1	1.568	8.712	0.004	0.080
Error	17.993	100	0.180			
Total	1473.407	103				
Corrected total	20.174	102				

The results of the ANCOVA suggested that group had a significant effect on students’ post-test level of intrinsic reading motivation (*F* = 8.712, *p* = 0.004) and the effect size was η^2^ = 0.080, which is a moderate effect. This effect size means that approximately 8% of the variance in post-test motivation scores is uniquely explained by the intervention, which is educationally meaningful. The pre-test level of intrinsic reading motivation had an effect on the post-test level (*F* = 3.187, *p* = 0.077), but did not have a significant effect. This suggested that, beyond the effect of initial intrinsic reading motivation, the implementation of intervention activity was the primary driver of the post-test differences. The insignificant pre-test effect further indicated that the observed post-test differences can be attributed to the intervention of Gen-AI integrated reading activity, rather than to pre-existing intrinsic reading motivation advantage.

### 4.2 Qualitative analysis

Seven students randomly selected from the experimental group participated in semi-structured focus group interviews. This interview lasted for about 30 min, asking students about how Gen-AI integrated science reading activity improve their intrinsic reading motivation. By analyzing the content discussed by students, the following themes were summarized:

1. Enhance students’ interest and engagement in science reading

In the interview, five students mentioned that Gen-AI integrated science reading activity can better enhance their interest in science reading:

*S3: I think I prefer the later way of reading activities. The previous way of reading activity is boring and uninteresting.*

*S1: Yes, I also think the use of Doubao makes reading more interesting. I am looking forward to reading activities every week now!*

*S2: I also feel that I am increasingly fond of reading science books now. Recently, I even bought a new science book to read!*

*S6: I also enjoyed the later reading activity, which allowed me to experience the joy of reading.*

*S4: Me too.*

This indicated that unlike the negative experiences of boredom in traditional reading activity mentioned by S3, Gen-AI allow students to freely ask their truly curious questions, transforming reading from completing tasks to satisfying curiosity and experiencing the joy of reading. Meanwhile, the viewpoint of S1 expressed students’ expectations for Gen-AI integrated reading activity, indicating that positive behavioral intentions have surpassed immediate emotions. In addition, the viewpoint of S2 showed that intrinsic motivation has generalized to science reading outside the classroom.

1. Improved understanding of reading materials

Three students mentioned that Gen-AI can provide specific and detailed explanations for science concepts they are not familiar with, enabling them to better understand reading materials:

*S7: I think the biggest help that Doubao has given me in reading is helping me better understand some science concepts in the reading materials. I remember when I was reading materials about the “greenhouse effect” on Earth, I actually did not have a clear understanding of the concept and consequences of the greenhouse effect. Then Doubao gave me an explanation, comparing carbon dioxide to a cotton jacket. I am the Earth, and wearing too many cotton jackets will definitely make me feel hotter and hotter. This made me understand the greenhouse effect immediately.*

*S6: Yes, I also think that Doubao helped me better understand some science concepts, especially concepts like photosynthesis and crustal movement, which are too abstract. At this point, Doubao will provide me with some examples in daily life or related videos, which is very helpful for me to understand these abstract concepts!*

*S2: Yes, I also feel that with the help of Doubao, I gain more knowledge from every reading activity than before. I think these knowledge are very helpful for my science learning.*

This indicated that the on-demand, real-time, and repeatable explanation function of Gen-AI solved the difficulty of teachers providing personalized concept understanding guidance for each student in traditional reading activities, enabling students to independently overcome comprehension barriers and experience experience the feeling of “I can learn” in science reading. This constituted an important mechanism for enhancing intrinsic reading motivation.

1. Inspire curiosity in science knowledge exploration

Two students mentioned the process of interacting with Gen-AI and proposing hypotheses at the beginning of the reading activity, which sparked their curiosity to explore science knowledge:

*S4: I particularly enjoy the game of “Guess” at the beginning of reading activities. Doubao has given us many hypotheses, asking us to choose which one is right. I definitely hope to win the game, so I hope to find relevant content in the reading materials.*

*S1: Yes, I also really enjoy this section. After selecting the answer, I quickly went to read the relevant materials. Every time I win a competition, I feel very happy and I feel like I’m starting to enjoy learning science knowledge more and more!*

This showed that, in the Gen-AI integrated reading activity, the Gen-AI facilitated introduction turned reading from passive to active (goal-directed engagement), the competence students felt from winning converted into enjoyment of science learning (success and emotional bonding), and the process of freely choosing among multiple hypotheses satisfied the need for autonomy.

1. Provide personalized reading guidance

Four students mentioned that Gen-AI can provide personalized guidance in reading activities, allowing them to understand the science knowledge they are interested in and want to know:

*S2: Compared to previous reading activities, I think the biggest change in experience is that I can learn about science knowledge that interests me from Doubao, and share this knowledge with other classmates.*

*S5: Yes, I feel the same way too. Previously, the reading questions were given to me by the teacher, and some of them were not actually of interest to me. Now I can find the questions I want to understand while reading, and then ask Doubao to explain them to me.*

*S1: During the group discussion, I found that we all raised a lot of questions about the reading materials, and maybe the teacher did not have time to answer each of our questions. At this point, Doubao became our second ‘teacher’!*

*S4: Yes, I feel the same way too!*

This indicated that personalized guidance enhanced intrinsic reading motivation through three pathways: autonomous question generation satisfied the need for autonomy, peer knowledge sharing fostered relatedness, and Gen-AI’s cognitive role-substitution protected students’ sense of competence by compensating for the limitation of teacher-student ratio and preventing questions from going unanswered. Among these, the metaphor of a “second teacher” highlighted students’ functional perception of Gen-AI as a pedagogical agent.

1. Enhance understanding of scientists’ research experiences

Three students mentioned that their virtual dialogues with “scientists” during the Gen-AI integrated reading activities provided them with a deeper understanding of scientists’ research experiences:

*S3: I particularly enjoy engaging in dialogues with scientists portrayed by Doubao. Previously, I was aware of certain scientists and the science knowledge they discovered, but I lacked a deep understanding of their research journeys. Through these dialogues, I have gained a deeper insight into the research experiences of these scientists.*

*S1: Yes, through this dialogue activity, I learned that the research journey of these scientists is not easy. They firstly proposed some hypotheses and then constantly used scientific methods to verify their hypotheses. They also encountered many difficulties during the research process, but they persisted. So these scientists are really great, and I also want to be a scientist in the future!*

*S5: I also think this dialogue activity is particularly good. It feels like these scientists are sharing their experiences with me like friends. I intend to continue learning learn more science knowledge in the future and apply it to solving more problems.*

This indicated that virtual dialogues with “scientists” role-played by Gen-AI enhanced intrinsic reading motivation through the following pathways: firstly, helping students gain a deeper understanding of the nature of science research. Secondly, facilitating students’ emotional internalisation from role-model identification to career aspirations, transforming relatedness into self-identity. Thirdly, enabling students’ role shift from listeners to active constructors, forming equal interaction and future behavioural intentions. Finally, through students’ autonomous choice of dialogue partners and questions, satisfying both autonomy and relatedness needs.

In interview with science teacher who participated in the activity, the teacher stated that the students in the experimental group had a more positive attitude towards science reading:

*In previous science reading activities, students would passively read after receiving the reading materials. But after the experimental group used Doubao, I felt the most obvious change was that they were more actively involved in reading than the control group students, and they would think and ask some very meaningful questions. For example, I remember during an activity, I assigned a reading material about “black holes,” and one group of students asked Doubao “Will the sun become a black hole?” This question I think is very valuable.*

Meanwhile, the teacher pointed out that integrating Gen-AI into science reading activity can provide guidance and assistance to students during reading process:

*Previously, when the whole class read the same content and discussed the same topics, children with poor foundations could not keep up, while those with good foundations found it boring. With Doubao, each group can ask questions and explore at their own pace. Groups with weaker levels can understand basic concepts, while groups with stronger levels can explore issues that interest them. This is something that traditional science reading activity find difficult to achieve.*

*In the past, when discussing, students would each speak their own way without a clear focus. With the integration of Doubao, students will first see how Doubao answers during group discussions, and then discuss whether this statement is correct or if there are other answers around Doubao’s answers. The quality of group presentations has also significantly improved. It’s a bit like Doubao building a ‘scaffold’ for them.*

However, the teacher also expressed that the design of Gen-AI integrated activity and the guidance of the teacher also play an important role in the implementation process:

*I think although using AI in science reading activities can be very helpful for students, there are also several points that need to be noted. Firstly, the design and management of the activity section are very important. In this experiment, I think the activity process was designed very well, especially the high participation of students in the dialogue with scientists played by AI. However, at the beginning, students were too curious and asked many questions unrelated to the reading topic, such as’ what do you like to eat ‘, which caused some classroom discipline to spiral out of control. Later on, we established questioning rules that required each group’s questions to be related to the current reading material, which improved the implementation of the activity.*

*The answers given by AI may not always be accurate. Sometimes it answers too abstractly, using many terms only learned in middle school or even high school, which students cannot understand at all and become even more confused. For example, once a student asked ‘why is the sky blue’, and AI answered ‘Rayleigh scattering’ and the relationship between light wavelength and particles, explaining it professionally, but it was too complicated for sixth grade students. At this point, I need to intervene immediately and explain it again in plain language. So I think AI is a great tool, but it can never replace teachers. When students should ask questions, when they should stop and summarize, and when they should bring the discussion back on track, all require teachers to judge and control.*

## 5 Discussion

Science reading is an important way to enhance the science literacy of primary school students and cultivate their positive attitude towards science learning (Schiefer et al., 2020). This study adopted a mixed method design to evaluate the effects of Gen-AI (Doubao) integrated science reading activity on primary school students’ intrinsic reading motivation. The quantitative results showed that students who receive Gen-AI integrated science reading activity had a more significant improvement in intrinsic reading motivation compared to traditional reading activity. Qualitative data generally supported this quantitative finding, with many students reporting positive attitudes and experiences towards Gen-AI integrated reading activity. These results align in direction with prior work suggesting that AI-based personalized reading or oral reading activities can promote intrinsic motivation (Alfaleh et al., 2025; Hidayat, 2024).

According to self-determination theory, when the three psychological needs of autonomy, competence and relatedness are met, individuals will have stronger intrinsic motivation and show higher engagement, persistence and satisfaction (Deci and Ryan, 2013; Ryan et al., 2021). In terms of autonomy, Gen-AI can provide students with the possibility of quickly generating multiple explanations and personalized reading guidance. Students can choose the direction of their questions based on their own interests and design interactions with Gen-AI. Many students mentioned in interviews that they can ask questions they are interested in and described their experience of leading questioning, suggesting that Gen-AI integrated reading activity may satisfy autonomy needs by increasing choice and control, thereby promoting the intrinsic reading motivation of students. In terms of competence, the instant and diverse explanations and personalized guidance that Gen-AI can provide in reading activity have provided some students with more direct cognitive support when understanding abstract concepts. In the qualitative report, some students expressed that through Gen-AI examples, they have understood science concepts that were previously incomprehensible, which is consistent with the quantitative results of improving intrinsic reading motivation, suggesting that the enhancement of competence may be one mechanism. In terms of relatedness, the discussion based on Gen-AI output, peer evaluation, and teacher feedback within the group formed an interactive context. Some students reported that Gen-AI integrated reading activity stimulated their curiosity to explore science knowledge, and simulation activities such as “conversations with scientists” gave them a deeper understanding of scientists’ research experiences. This indicates that Gen-AI integrated reading activity can under certain conditions foster relatedness.

However, the qualitative analysis showed that Gen-AI does not independently enhance all dimensions of the three needs, the teacher mentioned that answers given by Gen-AI sometimes are inaccurate, students may feel confused if there is a lack of teacher guidance, indicating that teacher played a key mediating role in this intervention, which is necessary for effective implementation but also complicates causal attribution. Thus, the observed effects are likely the result of interactions among Gen-AI, instructional design, and teacher facilitation rather than solely Gen-AI itself. To disentangle Gen-AI effects from teacher effects, future studies should adopt multi-teacher, multi-site designs, stricter randomization, or factorial experiments.

From a broader critical perspective, introducing Gen-AI raises issues of epistemic authority and information literacy. AI-generated text often adopts an authoritative tone but does not always provide verifiable sources or distinguish between established facts and hypotheses, without explicit guidance, students may develop excessive trust in AI statements, undermining their habits of critical evaluation and evidence checking (Melisa et al., 2025; Ncube et al., 2026). Therefore, AI should be positioned in the classroom as an assistive tool rather than an indisputable authority. Teachers must design activities that require students to check AI output against sources, compare evidence, and construct counterarguments. Such practices can both mitigate risks of AI misinformation and leverage AI affordances to foster information-literacy skills.

Based on the above, we offer several practical recommendations for integrating Gen-AI into primary school science reading activity: (1) explicitly frame Gen-AI as an assistive tool rather than an authoritative source, and routinely require source verification and evidence checking of Gen-AI outputs in class; (2) incorporate role assignments and structured evaluation rubrics to engage students in critical appraisal of AI-generated content, thus turning Gen-AI use into opportunities for metacognitive training; (3) provide teachers with training on prompt design, risk identification, and classroom moderation so they can effectively scaffold student-AI interactions while maintaining high-quality interpersonal engagement; and (4) retain and strengthen peer discussion and teacher feedback elements in curricula to balance Gen-AI convenience with the benefits of social learning.

## 6 Conclusion and limitations

This study designed an Gen-AI integrated science reading activity,and aimed to investigate its influence on primary school students’ intrinsic reading motivation. The quantitative results showed that compared with traditional science reading activity, Gen-AI integrated science reading activity significantly improved primary school students’ intrinsic reading motivation. The qualitative results provided the mechanism by which Gen-AI influences students’ intrinsic reading motivation, indicating that such activities promote the improvement of intrinsic reading motivation by enhancing students’ autonomy (such as providing personalized reading guidance), competence (such as improving understanding of reading materials), and relatedness (such as AI-generated peer discussions).

This study also has some limitations that should be addressed in future research. Firstly, this quasi-experimental study was conducted in a single school with one grade and one Gen-AI tool, which limits external validity. Secondly, this study adopted a quasi-experimental design. Although there was no significant difference between the two groups in the pre-test results, unobserved group differences or selection biases cannot be entirely ruled out (such as gender, science achievement, reading level, and prior experience with digital devices). Thirdly, the experimental group and control group in this study were organized by the same teacher. Although the teacher variable was controlled, teacher effects and potential Hawthorne effects (participants’ behavior changes due to being observed) may have influenced outcomes. Finally, the positive changes observed may be partly driven by novelty effects. Many students showed high interest and engagement when first exposed to Gen-AI, and this initial excitement could temporarily boost intrinsic motivation. These limitations imply that the findings of this study should be regarded as preliminary evidence, and future research with larger samples, multiple teachers, and longitudinal follow-up is needed to test the robustness and durability of these preliminary results.

## Data Availability

The raw data supporting the conclusions of this article will be made available by the authors, without undue reservation.

## References

[ref1] Ahmadi M. R. (2017). The impact of motivation on reading comprehension. Int. J. Res. Engl. Educ. 2, 1–7. doi: 10.18869/acadpub.ijree.2.1.1

[ref2] Alfaleh M. Albasis H. H. Mohamed A. M. (2025). EFL learners’ motivation and engagement with oral reading activities and AI tools: exploring the effect of gender, study level and English language proficiency. Cogent Educ. 12:2542395. doi: 10.1080/2331186x.2025.2542395

[ref3] Bandi A. Adapa P. V. S. R. Kuchi Y. E. V. P. K. (2023). The power of generative AI: a review of requirements, models, input–output formats, evaluation metrics, and challenges. Future Internet 15:260. doi: 10.3390/fi15080260

[ref4] Bommasani R. Hudson D. A. Adeli E. Altman R. Arora S. von Arx S. . (2021). On the opportunities and risks of foundation models. arXiv preprint arXiv:2108.07258. doi: 10.48550/arXiv.2108.07258

[ref5] Brindha R. Pongiannan R. Bharath A. Sanjeevi V. (2025). “Introduction to multimodal generative AI,” in Singh, A., and Singh, K. K., Eds., Multimodal Generative AI, (Singapore: Springer), 1–36.

[ref6] Brüggemann T. Ludewig U. Lorenz R. McElvany N. (2023). Effects of mode and medium in reading comprehension tests on cognitive load. Comput. Educ. 192:104649. doi: 10.1016/j.compedu.2022.104649

[ref7] Campbell D. T. Stanley J. C. (2015). Experimental and quasi-Experimental Designs for Research. Washington, DC: Ravenio books.

[ref8] Chan C. K. Y. Hu W. (2023). Students’ voices on generative AI: perceptions, benefits, and challenges in higher education. Int. J. Educ. Technol. High. Educ. 20:43. doi: 10.1186/s41239-023-00411-8

[ref9] Clarke V. Braun V. (2017). Thematic analysis. J. Posit. Psychol. 12, 297–298. doi: 10.1080/17439760.2016.1262613

[ref10] Cooper R. Fitzgerald A. Carpendale J. (2022). A reading group for science educators: an approach for developing personal and collective pedagogical content knowledge in science education. Int. J. Sci. Math. Educ. 20, 117–139. doi: 10.1007/s10763-022-10260-y, 30311153

[ref11] Dai D. W. Suzuki S. Chen G. (2025). Generative AI for professional communication training in intercultural contexts: where are we now and where are we heading? Appl. Linguist. Rev. 16, 763–774. doi: 10.1515/applirev-2024-0184

[ref12] Deci E. L. Ryan R. M. (2013). Intrinsic Motivation and Self-Determination in human Behavior. New York, NY: Springer Science & Business Media.

[ref13] Dewi P. K. Rofi'uddin A. Basuki I. A. (2025). How can gen-AI integration transform critical reading strategies? A case studies in Indonesian higher education. TPM – Test. Psychom. Methodol. Appl. Psychol. 32, 2092–2109.

[ref14] Duke N. K. Ward A. E. Pearson P. D. (2021). The science of reading comprehension instruction. Read. Teach. 74, 663–672. doi: 10.1002/trtr.1993

[ref15] Guthrie J. T. Coddington C. S. (2009). “Reading motivation,” in Wenzel, K. R., and Wigfield, A., (Eds.,) Handbook of Motivation at school, (New York, NY: Routledge), 517–540.

[ref16] He S. Lu Y. (2024). The effectiveness of gen AI in assisting students’ knowledge construction in humanities and social sciences courses: learning behaviour analysis. Interact. Learn. Environ. 32, 7041–7062. doi: 10.1080/10494820.2024.2415444

[ref17] Hidayat M. T. (2024). Effectiveness of AI-based personalised reading platforms in enhancing reading comprehension. J. Learn. Dev. 11, 115–125. doi: 10.56059/jl4d.v11i1.955

[ref18] Huang X. Lajoie S. P. (2023). Social emotional interaction in collaborative learning: why it matters and how can we measure it? Soc. Sci. Humanit. Open 7:100447. doi: 10.1016/j.ssaho.2023.100447

[ref19] Ismael S. A. Mohd O. AbdRahim Y. (2021). Cohen's kappa agreement among multiple raters in determining factors that influence MTUN academics on data sharing. Turk. J. Comput. Math. Educ. 12, 3766–3771.

[ref20] Jiang X. (2026). Explore the gen-AI empowerment black box: a meta-analysis of the impact of gen-AI on college students’ critical thinking. Interact. Technol. Smart Educ. 23, 514–545. doi: 10.1108/itse-01-2026-0004, 35579975

[ref21] Khlaif Z. N. Alkouk W. A. Salama N. Abu Eideh B. (2025). Redesigning assessments for AI-enhanced learning: a framework for educators in the generative AI era. Educ. Sci. 15:174. doi: 10.3390/educsci15020174

[ref22] Kline R. B. (2023). Principles and Practice of Structural Equation Modeling. New York, NY: Guilford publications.

[ref23] Koray Ö. Çetinkılıç S. (2020). The use of critical reading in understanding scientific texts on academic performance and problem-solving skills. Sci. Educ. Int. 31, 400–409. doi: 10.33828/sei.v31.i4.9

[ref24] Krueger R. A. Casey M. A. (2015). “Focus group interviewing,” in Handbook of Practical program Evaluation, eds. Newcomer K. E. Hatry H. P. Wholey J. S. (San Francisco, CA: Wiley), 506–534.

[ref25] Li H. Gan Z. (2022). Reading motivation, self-regulated reading strategies and English vocabulary knowledge: which most predicted students’ English reading comprehension? Front. Psychol. 13:1041870. doi: 10.3389/fpsyg.2022.1041870, 36591108 PMC9798900

[ref26] Liu D. (2024). The effects of segmentation on cognitive load, vocabulary learning and retention, and reading comprehension in a multimedia learning environment. BMC Psychol. 12:4. doi: 10.1186/s40359-023-01489-5, 38167380 PMC10759450

[ref27] Melisa R. Ashadi A. Triastuti A. Hidayati S. Salido A. Ero P. E. L. . (2025). Critical thinking in the age of AI: a systematic review of AI'S effects on higher education. Educ. Process: Int. J. 14:e2025031. doi: 10.22521/edupij.2025.14.31

[ref28] Mikk J. Kukemelk H. (2010). The relationship of text features to the level of interest in science texts. Trames. J. Humanit. Soc. Sci. 14:54. doi: 10.3176/tr.2010.1.04

[ref29] Ncube P. D. Dzvapatsva G. P. Matobobo C. Ranga M. M. (2026). Redefining student assessment in AI-infused learning environments: a systematic review of challenges and strategies for academic integrity. AI and Ethics 6:68. doi: 10.1007/s43681-025-00871-w

[ref30] Pescapè A. (2024). “Exploring the current state and future potential of generative artificial intelligence using a generative artificial intelligence,” in Santoianni, F., Giannini, G., and Ciasullo, A., (Eds.,) Mind, Body, and Digital Brains, (Cham: Springer), 37–56.

[ref31] Ramalingam K. Jiar Y. K. (2022). Influence of intrinsic and extrinsic motivation in learning among primary school students. Management 23:1889.

[ref32] Ravichandran P. Machireddy J. R. Rachakatla S. K. (2023). Data analytics automation with AI: a comparative study of traditional and generative AI approaches. J. Bioinform. Artif. Intell. 3, 168–190.

[ref33] Ryan R. M. Deci E. L. Vansteenkiste M. Soenens B. (2021). Building a science of motivated persons: self-determination theory’s empirical approach to human experience and the regulation of behavior. Motiv. Sci. 7, 97–110. doi: 10.1037/mot0000194

[ref34] Schiefele U. Schaffner E. Möller J. Wigfield A. (2012). Dimensions of reading motivation and their relation to reading behavior and competence. Read. Res. Q. 47, 427–463. doi: 10.1002/rrq.030

[ref35] Schiefer J. Golle J. Tibus M. Herbein E. Gindele V. Trautwein U. . (2020). Effects of an extracurricular science intervention on elementary school children's epistemic beliefs: a randomized controlled trial. Br. J. Educ. Psychol. 90, 382–402. doi: 10.1111/bjep.12301, 31353458 PMC7317376

[ref36] Sun L. Zhou L. (2024). Does generative artificial intelligence improve the academic achievement of college students? A meta-analysis. J. Educ. Comput. Res. 62, 1676–1713. doi: 10.1177/07356331241277937

[ref37] Troyer M. Kim J. S. Hale E. Wantchekon K. A. Armstrong C. (2019). Relations among intrinsic and extrinsic reading motivation, reading amount, and comprehension: a conceptual replication. Reading Writ. 32, 1197–1218. doi: 10.1007/s11145-018-9907-9

[ref38] Wang X.-W. Zhu Y.-J. Zhang Y.-C. (2022). An empirical study of college students’ reading engagement on academic achievement. Front. Psychol. 13:1025754. doi: 10.3389/fpsyg.2022.1025754, 36438359 PMC9687092

[ref39] Wigfield A. Guthrie J. T. (1997). Relations of children's motivation for reading to the amount and breadth or their reading. J. Educ. Psychol. 89:420. doi: 10.1037//0022-0663.89.3.420

